# Supplementary material for: A Novel Mast Cell Stabilizer JM25-1 Rehabilitates Impaired Gut Barrier by Targeting the Corticotropin-Releasing Hormone Receptors
Source: Pharmaceuticals (Basel). 2022 Dec 29;16(1):47. doi: 10.3390/ph16010047 (PMC9866683; doi:10.3390/ph16010047)
Supplement: Supplementary file 1 [file pharmaceuticals-16-00047-s001.zip › pharmaceuticals-2019520-supplementary.pdf]

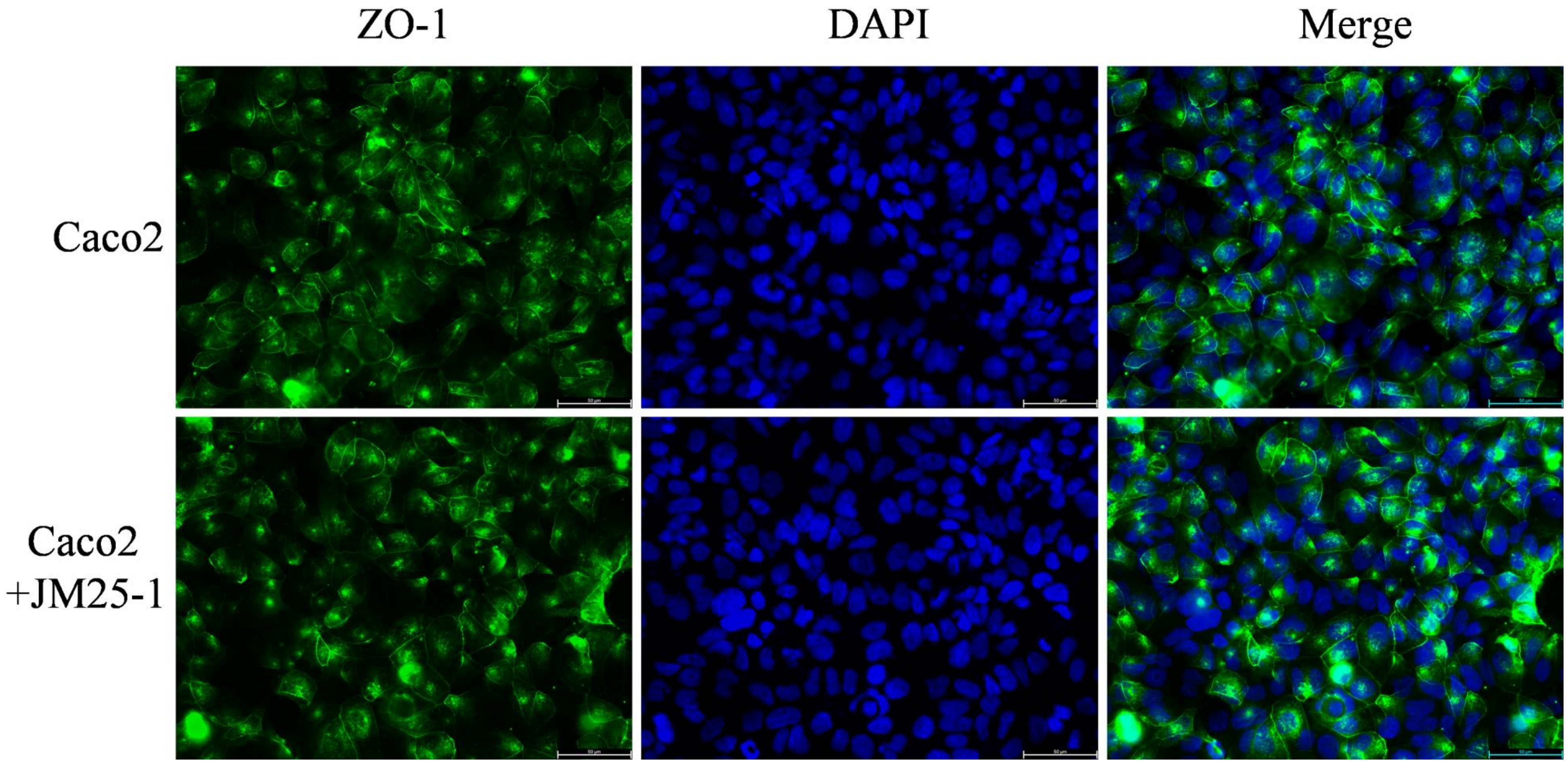

Figure S1: Effect of JM25-1 on tight junction in Caco2 cells. After treatment with JM25-1 for 24 h, immunofluorescence was used to observe ZO-1.

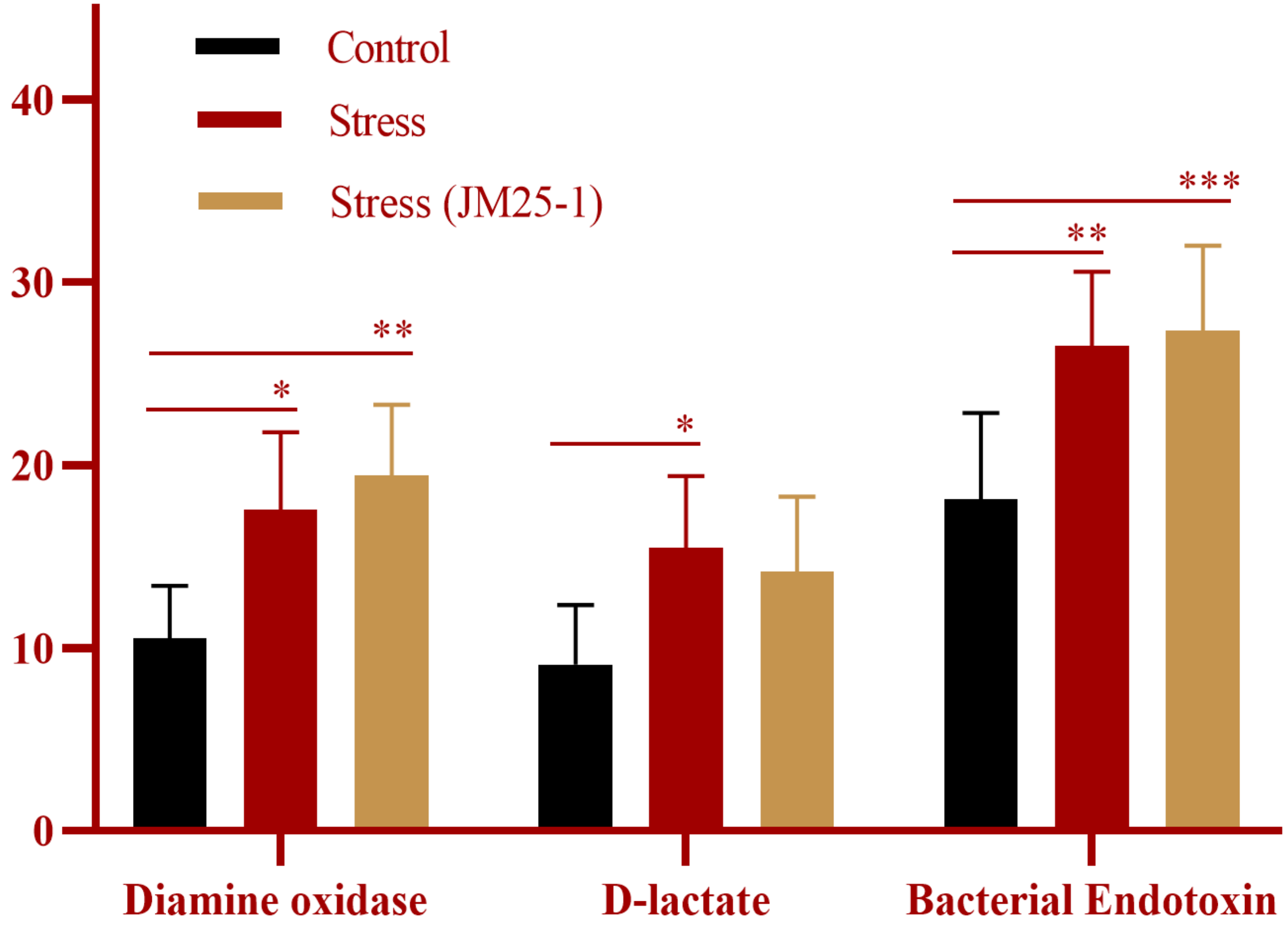

Figure S2: Evaluating the permeability of the stress model before JM25-1 treatment. The level of diamine oxidase, D-lactic acid and bacterial endotoxin in serum on day 7. bars, S.D. *$P \le 0.05$, **$P \le 0.01$; ***$P \le 0.001$.
